# Metacognitive awareness of the pretesting effect improves with self-regulation support

**DOI:** 10.3758/s13421-022-01392-1

**Published:** 2023-01-13

**Authors:** Steven C. Pan, Michelle L. Rivers

**Affiliations:** 1 Department of Psychology, Faculty of Arts and Social Sciences, College of Humanities and Sciences, National University of Singapore, Mailing address: 9 Arts Link, Singapore, 117572 Singapore; 2 Department of Psychology, University of California, Los Angeles, CA USA; 3 Department of Psychology, Texas Christian University, Fort Worth, TX USA; 4 Department of Psychological Sciences, Kent State University, Kent, OH USA

**Keywords:** Pretesting effect, Prequestions, Errorful generation, Metacognition, Knowledge updating

## Abstract

The *pretesting* or *prequestion effect* refers to the counterintuitive finding that taking tests on information that one has yet to learn, during which many erroneous responses typically occur, can benefit learning relative to nontesting methods (e.g., reading) if the correct answers are studied afterwards. Using a knowledge updating approach that entailed two or three cycles of pretesting versus reading followed by a criterial test, we investigated (a) the extent to which learners develop metacognitive awareness of the pretesting effect through experience (as evidenced by predictions of criterial test performance) and (b) three forms of external support—namely, *performance feedback* (displaying criterial test performance for pretested versus read items), *prediction reminders* (displaying learners’ predictions alongside performance feedback), and *recall prompts* (asking learners to remember criterial test performance during the first cycle prior to making predictions for the second cycle)—that might improve, or provide insights into, such awareness. Across five experiments, we found that learners generally lack awareness of the memorial benefits of pretesting, are predisposed to believing that reading is more effective even after repeatedly experiencing both techniques, and need support before they recognize that pretesting is more beneficial. Overall, these results underscore the challenge of, and highlight several means of dislodging, learners’ inaccurate beliefs about the efficacy of pretesting.

Taking a practice test on information that has yet to be learned might seem like a fruitless endeavor. After all, why bother attempting to answer test questions when there is almost no chance of producing the correct answers, and erroneous responses are produced instead? Remarkably, an emerging body of research reveals that engaging in such *pretesting* can improve memory substantially relative to nontesting methods (e.g., reading), provided that the correct answers are studied afterwards. This counterintuitive phenomenon, which is known as the *pretesting effect*, the *prequestion effect*, or the *errorful generation benefit*, has been demonstrated with word pairs and triplets (e.g., Huelser & Metcalfe, 2012; Pan et al., 2019), trivia facts (e.g., Kornell et al., 2009), text passages (e.g., Richland et al., 2009), and video lectures (e.g., Carpenter & Toftness, 2017), among other materials.

Several cognitive mechanisms have been implicated in the pretesting effect, including the generation of semantic mediators (i.e., words that link cues with targets), search set processes, error correction signals, reminding, and attentional factors (for a review, see Mera et al., 2021; see also Pan, Sana, Schmitt, et al., 2020; Potts & Shanks, 2014; Yang et al., 2017). By some accounts, taking a pretest activates mediators or candidate target words (forming a search set; i.e., a network of possible answers), facilitating later recall. Alternatively, generating incorrect answers may trigger an error correction signal (i.e., a neural learning process), and the incorrect response may itself become a retrieval cue for the correct answer. These mechanisms are not necessarily mutually exclusive, and theoretical research involving the pretesting effect remains ongoing.

Although some studies have found that the memorial benefits of pretesting are highly specific to the material that is directly pretested (e.g., James & Storm, 2019; Toftness et al., 2018), may require semantically relatedness between cues and targets (e.g., Huelser & Metcalfe, 2012; but see Potts & Shanks, 2014), and does not emerge in all cases (e.g., Geller et al., 2017), an emerging consensus is that the technique is competitive with better-established learning methods. For instance, in a recent study contrasting the efficacy of pretesting with retrieval practice (wherein learners take practice tests *after* having learned information), pretesting yielded better memory of encyclopedic text passages as evident on a criterial test conducted up to 48 hours later (Pan & Sana, 2021). Across four experiments, the pretesting condition exhibited a memory advantage of Cohen’s *d =* 0.30 over retrieval practice (i.e., small-to-medium size benefit), strengthening the conclusion that pretesting can be effective at enhancing learning.

## Metacognition of the pretesting effect

The pretesting literature implies that if learners adopt the technique during their learning activities, then substantial improvements will result. Models of self-regulated learning emphasize that decisions that learners make about the strategies to adopt during studying are informed by knowledge and beliefs about such strategies, often acquired through self-reflection on their performance (e.g., Efklides, 2011; Winne & Hadwin, 1998; Zimmerman, 2008; see also McDaniel & Einstein, 2020). If learners have the goal of improving their memory for material, but do not believe pretesting is effective for reaching that goal, then they are unlikely to engage in such a strategy spontaneously. The question follows: To what extent are learners metacognitively aware of the benefits of engaging in pretesting, and what approaches might be effective at fostering that awareness?

Initial studies have shown that learners remain unaware of the pretesting effect even after having the opportunity to use and benefit from the technique. For example, Huelser and Metcalfe (2012) had participants learn semantically related and unrelated cue–target word pairs using reading, in which pairs were presented intact (e.g., *bagel–breakfast*), or pretesting, in which the cue word was presented, and participants generated a response (e.g., *bagel–?*) before studying the pair intact. Next, participants took a criterial test assessing memory for those pairs, then rank ordered the efficacy of reading versus pretesting. Although pretesting yielded better memory than did reading (in the case of related pairs), participants consistently ranked pretesting as less effective. Similarly, Potts and Shanks (2014), Yang et al. (2017), and Zawadzka and Hanczakowski (2019) found that participants gave lower judgments of learning (JOLs; i.e., predict the likelihood of future recall) to pairs learned via pretesting versus reading when asked at the level of individual items and/or globally across all pretested or all read items (i.e., global-differentiated predictions).

Recent surveys of learners’ beliefs and practices (Pan, Sana, Samani, et al., 2020; Yang et al., 2017, Experiment 3) shed further light on the metacognitive unawareness of the pretesting effect. When asked to predict the relative effectiveness of pretesting versus reading in a hypothetical scenario, respondents tend to be agnostic or favor reading (44% of U.S. and Canadian student respondents and over 70% of online respondents in surveys have favored reading or studying). Further, students commonly endorse avoiding errors during learning, which is the opposite of what pretesting entails, plus use practice questions for retrieval practice more frequently than for pretesting (Pan, Sana, Samani, et al., 2020). Collectively, these results suggest that baseline beliefs towards pretesting are unfavorable and likely biased against the technique. That pattern is characteristic of many learners’ approaches towards “desirable difficulties”—that is, learning techniques that entail more effort and/or errors, at least during acquisition, but ultimately lead to longer-lasting learning (for further discussions, see Bjork, 1994; Bjork et al., 2013; Rivers, 2021).

What approaches, then, might be effective at reversing learners’ metacognitive unawareness of the pretesting effect? Having a single experience with pretesting, as in the case of Huelser and Metcalfe (2012) and other studies, appears to be insufficient. Alternatively, one might directly inform learners about the pretesting effect: Yang et al. (2017); Experiment 4) had participants read about pretesting prior to using pretesting and reading to learn a series of word pairs. Relative to a condition that did not receive such information, that approach yielded higher global JOLs and equivalent item-level JOLs for pretested versus read items. Although mixed, these results reveal malleability in learners’ metacognitive beliefs about pretesting, plus raise the possibility that other approaches might yield further improvements in those beliefs.

## Fostering awareness of effective learning strategies through task experience

The current study explored whether having learners experience pretesting and reading more than once—that is, across multiple training–test cycles as opposed to once as in prior research—facilitates metacognitive awareness of the pretesting effect. Practically, direct experience with various strategies may be more effective than instructional interventions at convincing learners that a given strategy is effective *for them* and not just learners in general (Koriat & Bjork, 2006; McDaniel & Einstein, 2020; Yan et al., 2016). If extended experience does so for the case of pretesting, then that would suggest an important step towards promoting students’ self-regulated use of effective strategies.

The method of *knowledge updating*—that is, learning about the relative effectiveness of different strategies from task experience (first introduced by Brigham & Pressley, 1988)—informed the development of this study. According to Dunlosky and Hertzog’s (2000) knowledge updating framework, four critical assumptions must be met in order for knowledge updating to occur: (a) one strategy must be more effective than another at improving memory (the effectiveness assumption); (b) learners must become aware of the differential strategy effectiveness via monitoring behaviors during task activity or on a subsequent test (the monitoring assumption); (c) learners must attribute those differences to the specific strategies that were used (the updating assumption); and (d) learners must use their newly acquired knowledge when making new metacognitive judgments (the utilization assumption). Although we expected that pretesting would easily satisfy the first assumption, we expected that meeting the remaining assumptions would be more challenging given the cognitive demands for each case. Consistent with that possibility, task experience alone can be insufficient for learners to correct inaccurate beliefs about learning techniques (e.g., Hertzog et al., 2009; Matvey et al., 2002; Mueller et al., 2015; Price et al., 2008; Tullis & Benjamin, 2012). Various forms of support, however, have been shown to promote knowledge updating for other effective learning strategies (e.g., Mueller et al., 2015; Price et al., 2008; Rivers et al., 2022; Yan et al., 2016).

For example, Tullis et al. (2013); Experiments 2–4) had learners study a set of word pairs, practice the pairs using retrieval practice or restudying, make global predictions of future test performance (for pairs learned using each of the two techniques), take a 1-day delayed criterial test on those pairs, and then repeat the procedure with new pairs. Although most participants’ criterial test performance was higher for pairs practiced using retrieval versus restudy, both the initial and subsequent predictions did not reflect this performance advantage (Experiment 2). One proposed explanation for the lack of knowledge updating was that learners faced a heavy metacognitive burden during the learning task and had difficulty tracking the number of pairs practiced using retrieval versus restudy (i.e., a failure to meet the monitoring assumption). To overcome that burden, subsequent experiments added external support in the form of feedback on the final criterial test. Knowledge updating improved with feedback about the technique with which each pair had originally been learned (Experiment 3), and even further with global feedback about criterial test performance on all tested versus restudied pairs (Experiment 4). Once the monitoring assumption was met—that is, by providing learners with feedback so they did not have to track performance themselves—learners were able to update their knowledge about strategy effectiveness.

The results of Tullis et al. (2013) and other knowledge updating studies underscore the considerable support that may be needed to help learners overcome misperceptions about desirable difficulties and other evidence-based learning techniques. On that basis, we anticipated that similar approaches might be needed to foster knowledge updating about the pretesting effect. Although the knowledge updating framework does not specifically predict which forms of self-regulation support (i.e., scaffolds) will be effective, we incorporated several support methods aimed at addressing assumptions of the framework.

## The current study

Within each of four experiments, participants completed a first cycle wherein they learned pairs using pretesting or reading, made global predictions of future test performance for pretested and read items, and then took a criterial test. After a 5-minute delay, they repeated these steps during a second cycle using new pairs (a fifth experiment added a third cycle). Given prior research, we did not expect participants to exhibit awareness of the pretesting effect during the first cycle. The critical question was whether any knowledge updating would manifest during the second cycle, and if not, whether different forms of external support—all drawing on assumptions of the knowledge updating framework—might be effective at facilitating such updating.

## Experiment 1

The first experiment investigated whether repeated firsthand experience with pretesting and reading facilitates metacognitive awareness of the benefits of pretesting.

### Method

All experiments in the study were programmed using the open-source platform Collector (Garcia, 2015). Experiment 1 was preregistered (https://osf.io/pwnr2).

#### Participants

The target sample size for Experiments 1–4 was 53 participants (cf. Tullis et al., 2013), which according to an a priori power analysis conducted in G*Power (Faul et al., 2007) should yield 80% power to detect an effect size of Cohen’s *d =* 0.35 or larger (based on a one-tailed, one-sample *t* test with α = 0.05). We posted slots exceeding that amount on Amazon Mechanical Turk and provided compensation of USD $2.75 per participant. All participants were from North America, fluent in English, and had an approval rate of 95% or higher on the platform. Data were analyzed from the 47 participants (*M*_age_ = 35.4 years, 66% male) who completed the experiment without technical issues and submitted a valid completion code (in Experiments 1–4, just enough participants were recruited to exceed the sample size target; Experiment 5 featured a larger sample for better statistical power). A further 22 participants were dropped due to noncompliance with instructions. Experiments 1–4 were approved by the Institutional Review Boards of the University of California, Los Angeles, and Kent State University.

#### Design

Experiment 1 used a 2 (cycle: 1 vs. 2) × 2 (practice condition: read vs. pretested) within-participants design.

#### Materials

The materials consisted of four lists containing 16 word pairs each (from Huelser & Metcalfe, 2012; drawn from Nelson et al., 1998, norms). Each pair featured two words of ≥4 letters each, with forward and backward associative strengths (i.e., the likelihood of one word to elicit recall of the other) of 0.05–0.054 and 0, respectively.

#### Procedure

**Fig. 1:**
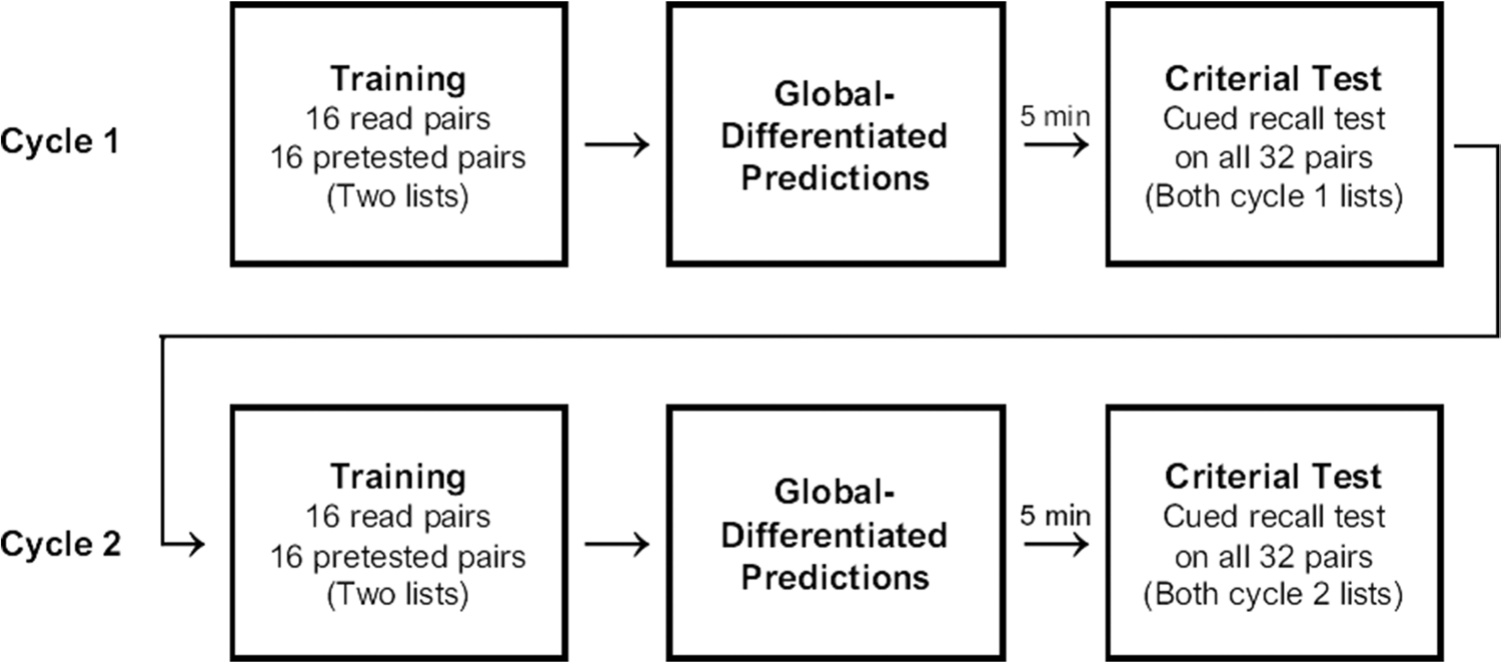
Overview of experimental procedure. *Note.* Within each of two consecutive cycles, participants learned 32 word pairs via reading or pretesting (two lists of 16 pairs each, randomly intermixed), made global-differentiated predictions, and then took a 5-min delayed criterial test. Experiments 2–4 featured performance feedback after both criterial tests. The feedback in the first cycle of Experiments 3–4 also included reminders of participants’ original predictions. During the second cycle of Experiment 4, participants were further prompted to recall their test performance in Cycle 1 prior to making new predictions. Experiment 5 featured the same single-session, multicycle design but included three cycles instead of two

The procedure is summarized in Fig. 1. During each of two cycles, participants learned a list of pairs via pretesting or reading, made global-differentiated predictions, and then completed a 5-minute delayed criterial test. Assignment of lists to practice condition and cycle were counterbalanced. A video of the procedure is available at https://osf.io/24efh.

##### Cycle 1

Participants first read instructions stating that they were to learn a series of pairs using reading (“*Please read so that you remember the word pair well*”) or pretesting (“*You will be shown the first word and a text box. You will have 5 seconds to type the missing word into the box. Please think of what the missing word might be, and type your answer as quickly as possible.*”). Thirty-two pairs (i.e., two complete lists) were then presented, half via reading and the other half via pretesting. Each pair appeared one a time and in random order (i.e., pretested and read pairs were randomly intermixed). As in Huelser and Metcalfe (2012), the read condition involved the presentation of a given pair in its entirety for 5 s each, whereas in the pretested condition, the first word of a given pair was presented for 5 s, during which participants entered their guess for the second word, and after which the entire pair was presented for an additional 5 s.^1^

Next, participants made global-differentiated predictions for reading and pretesting. The predictions occurred in random order (reading or pretesting first) and in response to the following prompt: “*There were 16* read (pretested) *word pairs. If you were to be tested (shown the first word and have to recall the second) on those 16* read (pretested) *word pairs approximately 5 minutes from now, how many do you think you would answer correctly?*” Allowed responses ranged from 0 to 16.

After a 5-min distractor task (involving the game Tetris), participants completed a self-paced criterial test that assessed memory for each of the 32 pairs that had been learned. On each test trial, the first word of a given pair was shown, and the missing word had to be typed. All pairs were tested one at a time, in random order, and without feedback.

##### Cycle 2

Immediately after Cycle 1, participants engaged in an identical set of tasks as in Cycle 1 but involving 32 new pairs. Further, after the criterial test, participants answered three exit questions that addressed (a) whether they would prefer to use reading or pretesting to learn a new list of pairs and (b) how effective they believed reading and pretesting are for helping one learn and remember information (answered on a 0–10 scale, with anchors ranging from *utterly ineffective* to *completely effective*). The experiment concluded afterwards.

### Results and discussion

All statistical tests reported in this manuscript are two-tailed (although we hypothesized that any observed differences in test performance and predictions would favor pretesting, we opted for the more cautious approach of using two-tailed tests). To supplement null-hypothesis significance testing, for all *t* tests we also report Bayes factors (calculated using the *BayesFactor* package in R; Morey et al., 2022), which are defined as the ratio of the likelihood of the data given the alternative hypothesis to the likelihood of the data given the null hypothesis (*BF*_*10*_). A *BF*_*10*_ greater than 1 suggests that the alternative hypothesis is more likely, a *BF*_*10*_ of 1 suggests that both hypotheses are equally likely, and a *BF*_*10*_ less than 1 suggests that the null hypothesis is more likely (for discussion, see Rouder et al., 2009; Wagenmakers, 2007). In cases where the null hypothesis is more likely, Bayes factors are reported as the reciprocal *BF*_*01*_ for ease of interpretation. Effect sizes for *t* tests are reported in terms of Cohen’s *d* (i.e., *d*_z_, for one-sample comparisons; Lakens, 2013).

**Fig. 2:**
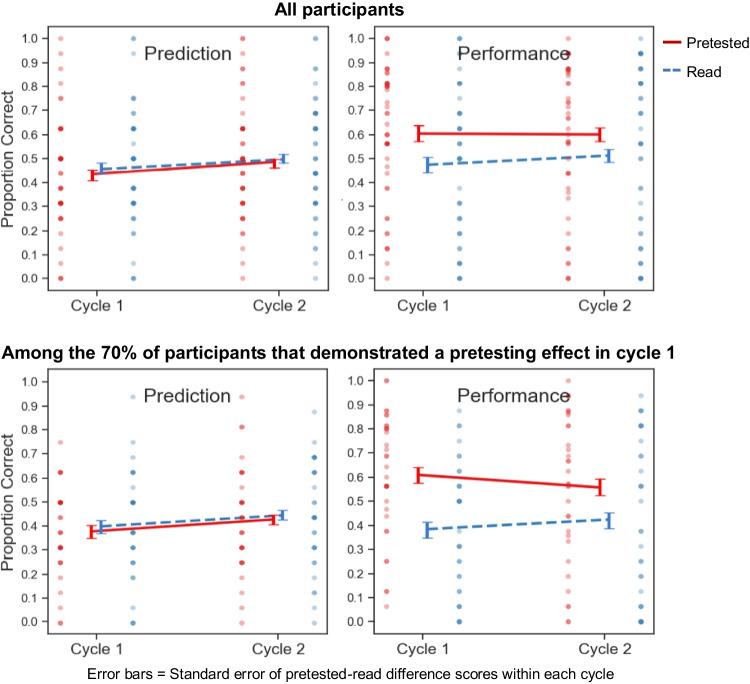
Global-differentiated predictions and criterial test performance in Experiment 1

Global-differentiated predictions and criterial test results for Experiment 1 are depicted in Fig. 2 (left- and right-side panels, respectively). The top row displays data from all participants, whereas the bottom row displays data from participants that exhibited a numerical pretesting effect in Cycle 1.

#### Initial test performance

As was expected given no prior knowledge of the pairs, participants rarely generated the correct answer to pretest trials in Cycle 1 (*M* = .029, *SE* = .0065) or in Cycle 2 (*M* = .032, *SE* = .00073).

#### Criterial test performance

In this and all subsequent experiments, following Huelser and Metcalfe (2012), criterial test trials in which the correct answer had been successfully guessed (accounting for no more than .053 of all pretested pairs throughout the study) were excluded from analysis. Participants recalled a greater proportion of pretested pairs in Cycle 1, *t*(46) = 3.92, *p* = .00029, *d* = 0.57, *BF*_*10*_ = 88.43, and in Cycle 2, *t*(46) = 3.18, *p* = .0027, *d* = 0.46, *BF*_*10*_ = 12.20.

#### Global-differentiated predictions

We conducted a 2 (cycle: 1 vs. 2) × 2 (practice condition: read vs. pretested) repeated-measures analysis of variance (ANOVA) on global-differentiated predictions. Full outcomes of that ANOVA are reported in Table 1. The main effect of cycle, main effect of practice condition, and their interaction were not significant. When we restricted our analysis to the 70% of participants that demonstrated a numerical pretesting effect on the Cycle 1 criterial test (necessary to meet the effectiveness assumption of the knowledge updating framework), the same pattern held, with a nonsignificant interaction between cycle and practice condition, *F*(1, 32) = 1.60, *p* = .22, η_p_^2^ = 0.048.

**Table 1:** Outcomes of analyses of variance for globally differentiated predictions in Experiments 1–4

	*df*	*F*	*p*	η_p_^2^
Experiment 1: 2 (cycle: 1 vs. 2) × 2 (practice condition: read vs. pretested)				
Main effect of cycle	1, 46	2.64	.11	0.54
Main effect of practice condition	1, 46	<0.01	>.99	<0.01
Interaction	1, 46	1.30	.26	0.027
Experiment 2: 2 (cycle: 1 vs. 2) × 2 (practice condition: read vs. pretested)				
Main effect of cycle	1, 49	8.55	.0052**	0.026
Main effect of practice condition	1, 49	0.074	.79	0.0015
Interaction	1, 49	5.22	.027*	0.0064
Experiment 3: 2 (cycle: 1 vs. 2) × 2 (practice condition: read vs. pretested)				
Main effect of cycle	1, 51	8.83	.0045**	0.15
Main effect of practice condition	1, 51	<0.01	.97	<0.01
Interaction	1, 51	10.92	.0017**	0.18
Experiment 4: 2 (cycle: 1 vs. 2) × 2 (practice condition: read vs. pretested)				
Main effect of cycle	1, 48	1.29	.26	0.026
Main effect of practice condition	1, 48	3.60	.064	0.070
Interaction	1, 48	10.32	.0024**	0.18

*Note.* * and ** indicate *p* values < .05 and .01, respectively

As shown in Fig. 2, no significant difference was found between participants’ predictions for read and pretested pairs in Cycle 1, *t*(32) = 0.35, *p* = .73, *d* = 0.061, *BF*_*01*_ = 5.07, or in Cycle 2, *t*(32) = 1.83, *p* = .076, *d* = 0.32, *BF*_*01*_ = 1.20. Thus, learners did not spontaneously develop awareness of the pretesting effect through experience.

#### Judgments of reading and pretesting

Table 2 reports the number of participants that preferred pretesting and reading, respectively, for all experiments. Ratings of the effectiveness of reading (*M* = 6.87, *SE* = .33) and pretesting (*M* = 6.43, *SE* = .37) did not significantly differ, *t*(46) = 1.00, *p* = .32, *d* = 0.15, *BF*_*01*_ = 3.93.

**Table 2:** Preference for reading versus pretesting in Experiments 1–5

	Reading	Pretesting
Experiment 1	60%	40%
Experiment 2	49%	51%
Experiment 3	48%	52%
Experiment 4	43%	57%
Experiment 5, performance feedback	25%	75%
Experiment 5, performance feedback with reminders	25%	75%

## Experiment 2

The lack of significant knowledge updating in Experiment 1 implies that metacognitive awareness of the pretesting effect may require external support to develop. Accordingly, in Experiment 2 we added individualized *performance feedback—*that is, feedback revealing how many pairs were correctly recalled in the read and pretested conditions on the criterial test. Given that such feedback removes the need to remember which technique had been used for each word pair, which may have limited knowledge updating due to a failure to meet the monitoring assumption (cf. Bieman-Copland & Charness, 1994; Hertzog et al., 2009; Mueller et al., 2015; Price et al., 2008), we predicted that its use would lead to greater updating. Experiment 2 was further motivated by evidence that individualized feedback can enhance metacognitive awareness of relative strategy effectiveness (at least for retrieval practice versus restudy, as in Hui et al., 2021; Tullis et al., 2013).

### Method

Experiment 2 was preregistered (https://osf.io/2xujs).

#### Participants

Due to concerns over incomplete responses in Experiment 1 and potential data quality issues during the COVID-19 pandemic (e.g., Lee & Hoffman, 2020; see also Kennedy et al., 2020), the remainder of the study was conducted using the Prolific Academic platform (which has additional data quality controls; Palan & Schitter, 2018). Each participant was in North America, Australia, New Zealand, or the United Kingdom; fluent in English; had an approval rate of 90% or higher on prior Prolific studies; and received compensation of USD $4.75. Data were analyzed from the 50 participants (*M*_age_ = 32.8 years, 55% female) that completed the entire experiment and submitted a valid completion code. An additional six participants were excluded due to failing an attention check.

#### Design, materials, and procedure

**Fig. 3:**
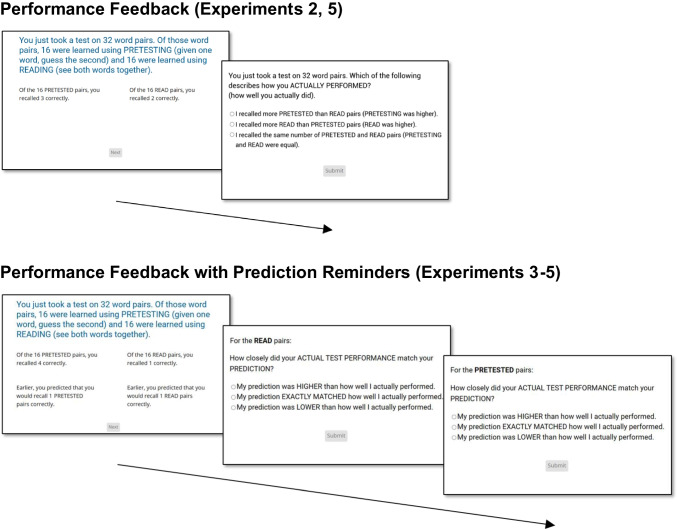
Performance feedback (Experiments 2–5) and feedback with prediction reminders (Experiments 3–5). *Note.* The above screenshots display the different forms of external support that appeared after the criterial test in the first cycle of the indicated experiment (and, in Experiment 5, after the second cycle as well). After viewing performance feedback (or performance feedback with prediction reminders), participants were asked to indicate the performance difference between the read and pretested conditions (Experiment 2 and the performance feedback group in Experiment 5), or the degree to which their prediction matched their actual performance in each of the read and pretested conditions (Experiments 3, 4, and the performance feedback with prediction reminders group of Experiment 5). In Experiments 3–5, such feedback, where provided, was viewed once more before beginning the next cycle of the experiment. See https://osf.io/jxwcr for a larger version of this figure

Experiment 2 was identical to Experiment 1 except for the following changes. First, performance feedback was provided after both criterial tests. That feedback, which is depicted in the top row of Fig. 3, took the form of a screen that displayed the number of pairs (out of 16) that had been correctly recalled in the read and pretested conditions. To facilitate comparison, those scores were displayed side by side. Further, in Cycle 1, participants were subsequently asked to identify whether their performance was higher in the pretested versus read condition, the read versus pretested condition, or equal in both conditions. That question was included to reinforce the feedback and served as an attention check (wherein an inaccurate response resulted in removal from the study).

In Cycle 2, feedback was not reinforced or subject to an attention check. Instead, to further probe the extent to which the updating assumption of the knowledge updating framework had been met, we asked participants to attribute their criterial test performance to one of five options (e.g., differential effectiveness of learning method, lucky guesses, and an “other” option; see Table 3 for full details).

**Table 3:** Attributions for differences in criterial test performance between read and pretested items in Experiments 2–5

	Differential effectiveness of learning method	Pairs were easier in one condition	I made lucky guesses	Both methods were equally effective	Other
Experiment 2	67%	12%	8%	8%	4%
Experiment 3	58%	21%	6%	10%	6%
Experiment 4	65%	6%	8%	16%	4%
Experiment 5, performance feedback	74%	13%	1%	6%	5%
Experiment 5, performance feedback with reminders	72%	8%	8%	10%	3%

### Results and discussion

**Fig. 4:**
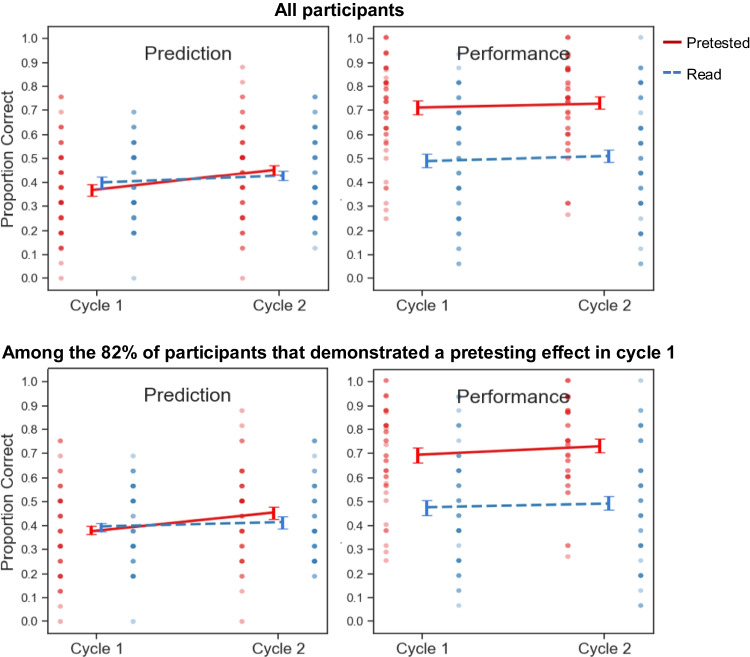
Global-differentiated predictions and criterial test performance in Experiment 2

Global-differentiated prediction and criterial test data are depicted in Fig. 4.

#### Initial test performance

As in Experiment 1, participants infrequently generated the correct answer to pretest trials in Cycle 1 (*M* = .035, *SE* = .0074) or in Cycle 2 (*M* = .040, *SE* = .0065).

#### Criterial test performance

In Cycle 1, participants recalled a greater proportion of pretested pairs, *t*(49) = 7.68, *p* < .0001, *d* = 1.09, *BF*_*10*_ > 100. In Cycle 2, participants recalled a greater proportion of pretested pairs, *t*(49) = 8.52, *p* < .0001, *d* = 1.20, *BF*_*10*_ > 100.

#### Global-differentiated predictions

A repeated-measures ANOVA analogous to that performed for Experiment 1 revealed a significant interaction between cycle and practice condition, *F*(1, 49) = 5.22, *p* = .027, η_p_^2^ = 0.0064, suggesting that predictions for reading and pretesting differed across cycles (see Table 1 for full results). When analysis was restricted to the 82% of participants that showed a pretesting effect in Cycle 1, a significant interaction was also observed*, F*(1, 40) = 4.19, *p* = .047, η_p_^2^ = 0.0074. Those results suggest that performance feedback was effective at eliciting knowledge updating.

Visual inspection of the results (see Fig. 4), however, reveals that knowledge updating was marginal at best. Indeed, a pair of follow-up *t* tests involving data from participants that demonstrated a pretesting effect in Cycle 1 revealed no significant difference between pretested and read predictions in Cycle 1, *t*(40) = 1.17, *p =* .25, *d =* 0.18, *BF*_*01*_ = 3.15, or in Cycle 2, *t*(40) = 1.60, *p =* .12, *d =* 0.25, *BF*_*01*_ = 1.84. Hence, performance feedback was only partially effective at dislodging learners’ inaccurate perceptions about the effectiveness of pretesting versus reading.

#### Judgments of reading and pretesting

Ratings of the effectiveness of reading (*M* = 6.02, *SE* = .34) and pretesting (*M* = 6.22, *SE* = .35) did not significantly differ, *t*(48) = 0.40, *p* = .69, *d* = 0.057, *BF*_*01*_ = 5.97.

#### Attributions for criterial test performance

Table 3 reports participants’ attributions for a difference in performance between conditions (for Experiment 2 and all subsequent experiments), the most common of which was a differential effectiveness of learning method.

## Experiment 3

In Experiment 2, directly informing learners of their criterial test performance presumably helped meet the monitoring assumption of the knowledge updating framework. Yet doing so was only partially successful at inducing changes in global-differentiated predictions. That result suggested a need for more extensive support. Accordingly, in Experiment 3, we implemented performance feedback with *prediction reminders*—that is, feedback that included criterial test performance and original predictions displayed simultaneously—plus asked participants to contrast their predictions with their actual performance.

Precedent for improved metacognition following self-examination of performance-prediction discrepancies comes from research on classroom calibration (e.g., Hacker et al., 2000; Miller & Geraci, 2011; Saenz et al., 2017; for a review, see Hacker & Bol, 2019). For example, Hacker et al. (2000) had college students predict exam performance during a psychology course, then returned the exams after they had been graded. The students compared their predictions with their exam performance and reflected on any discrepancies; doing so yielded increasingly accurate predictions across three successive exams. By fostering reflection on the reasoning used to make metacognitive judgments, the prediction reminders in Experiment 3 might yield similar improvements. We hypothesized that calling attention to prediction-performance discrepancies would facilitate fulfillment of the updating and (especially) the utilization assumptions of the knowledge updating framework, leading to improved metacognitive accuracy.

### Method

Experiment 3 was preregistered (https://osf.io/64x5k).

#### Participants

Participants were recruited using the Prolific Academic platform in the same manner as in Experiment 2. Data were analyzed from the 52 participants (*M*_age_ = 34.6 years, 66% female) that completed the entire experiment and submitted a valid completion code. An additional eight participants were excluded due to failing attention checks.

#### Design, materials, and procedure

Experiment 3 was patterned after Experiment 2, except for the following modifications. First, the feedback after the criterial test in Cycle 1 included not just performance for pretested and read pairs but also the earlier global-differentiated predictions for each condition, presented side by side. That feedback is depicted in the bottom row of Fig. 3. Participants were told to check how closely their prediction matched their actual performance. Second, participants answered two follow-up multiple-choice questions, one each for the read and pretested conditions and displayed on separate screens, in which they had to contrast their predictions and actual performance (e.g., “My prediction was higher than how well I actually performed”; see Table 4 for full details). Both questions also served as attention checks in the same manner as in Experiment 2. Finally, participants were required to view the feedback once more before beginning Cycle 2.

**Table 4:** Performance-prediction discrepancies in Experiments 3–5

			Relative to performance, prediction was…		
	Cycle	Condition	Lower	Higher	Same
Experiment 3	1	Read	63%	31%	6%
	1	Pretested	76%	15%	6%
Experiment 4	1	Read	56%	36%	6%
	1	Pretested	80%	16%	2%
Experiment 5, performance feedback with reminders	1	Read	69%	21%	10%
	1	Pretested	82%	15%	3%
	2	Read	47%	34%	19%
	2	Pretested	81%	12%	8%

Overall, the feedback implemented in cycle 1 was intended to ensure that participants not only were aware of their criterial test performance, but also whether their performance reflected their earlier predictions. The remainder of the experiment, including the feedback given at the end of Cycle 2, was unchanged relative to Experiment 2.

### Results and discussion

**Fig. 5:**
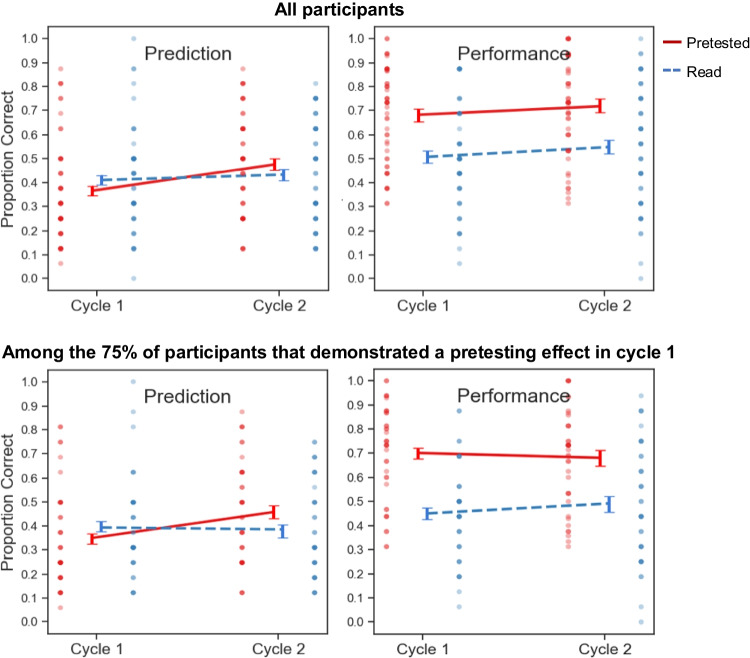
Global-differentiated predictions and criterial test performance in Experiment 3

Global-differentiated prediction and criterial test data are depicted in Fig. 5.

#### Initial test performance

As in prior experiments, participants rarely generated the correct answer to pretest trials in Cycle 1 (*M* = .035, *SE* = .0060) or in Cycle 2 (*M* = .053, *SE* = .0067).

#### Criterial test performance

In Cycle 1, participants recalled a greater proportion of pretested pairs, *t*(51) = 6.67, *p* < .00001, *d* = 0.93, *BF*_*10*_ > 100. In Cycle 2, participants recalled a greater proportion of pretested pairs, *t*(51) = 5.99, *p* < .0001, *d* = 0.83, *BF*_*10*_ > 100.

#### Global-differentiated predictions

A repeated-measures ANOVA on global-differentiated predictions analogous to that performed for the prior experiments revealed a significant interaction between cycle and practice condition, *F*(1, 51) = 10.92, *p* = .0017, η_p_^2^ = 0.18 (see Table 1 for full results). When analysis was restricted to the 75% of participants that showed a pretesting effect on the Cycle 1 criterial test, the interaction between cycle and practice condition remained significant, *F*(1, 38) = 18.23, *p* = .00013, η_p_^2^ = .32.

As is evident in Fig. 5, participants updated their Cycle 2 predictions to reflect an advantage of pretesting over reading and did so to a more pronounced extent than in prior experiments, although such updating remained well short of the actual magnitude of the observed pretesting effect. Follow-up *t* tests performed on the predictions made by participants that demonstrated a pretesting effect in Cycle 1 confirmed that successful updating had occurred: In Cycle 1, those participants made higher predictions for read pairs, *t*(38) = 2.11, *p =* .042, *d =* 0.34, *BF*_*10*_ = 1.24, but that pattern was reversed in cycle 2, with higher predictions for the pretested pairs, *t*(38) = 2.55, *p =* .015, *d =* 0.41, *BF*_*10*_ = 2.94.

#### Performance-prediction discrepancies

Table 4 reports, for Experiment 3 and all subsequent experiments, the percentage of participants who gave predictions that were lower, higher, or exactly matching performance in the pretested and read conditions.

#### Judgments of reading and pretesting

Ratings of the effectiveness of reading (*M* = 5.87, *SE* = .31) and pretesting (*M* = 6.35, *SE* = .28) did not significantly differ, *t*(51) = 1.19, *p* = .24, *d* = 0.17, *BF*_*01*_ = 3.40.

## Experiment 4

In Experiment 3, performance feedback coupled with calling learners’ attention to discrepancies between their predictions and test performance fostered awareness of the benefits of pretesting. Experiment 4 sought to replicate that finding and explore the extent to which memory for Cycle 1 test performance (as solicited via a *recall prompt* that was presented just prior to the prediction-making step in Cycle 2) is predictive of successful knowledge updating. The recall prompt allowed us to examine whether the utilization assumption of the knowledge updating framework, which posits that learners have firsthand knowledge about the effectiveness of learning strategies that they have experienced, was met by ensuring that learners remembered the feedback that was provided during the Cycle 1 criterial test (cf. Mueller et al., 2015). Moreover, the recall prompt itself might lead to even greater updating than in prior experiments by making memory for Cycle 1 test performance more salient.

### Method

Experiment 4 was preregistered (https://osf.io/f63c5).

#### Participants

Participants were recruited using the Prolific Academic platform in the same manner as in prior experiments. Data were analyzed from the 49 participants (*M*_age_ = 32.3 years, 53% male) that completed the entire experiment and submitted a valid completion code. An additional five participants were excluded due to failing attention checks.

#### Design, materials, and procedure

Experiment 4 was identical to Experiment 3, except for the following changes. In Cycle 2, immediately prior to making global-differentiated predictions, participants were prompted to recall the exact number of pairs that they had successfully recalled in the read and pretested conditions in Cycle 1. After entering both numbers, they were further asked to recall whether their performance had been higher in the pretested versus read conditions, higher in the read versus pretested conditions, or equal in both conditions. The purpose of these questions was to record whether participants could remember—either exactly or more generally—their performance in Cycle 1.

### Results and discussion

**Fig. 6:**
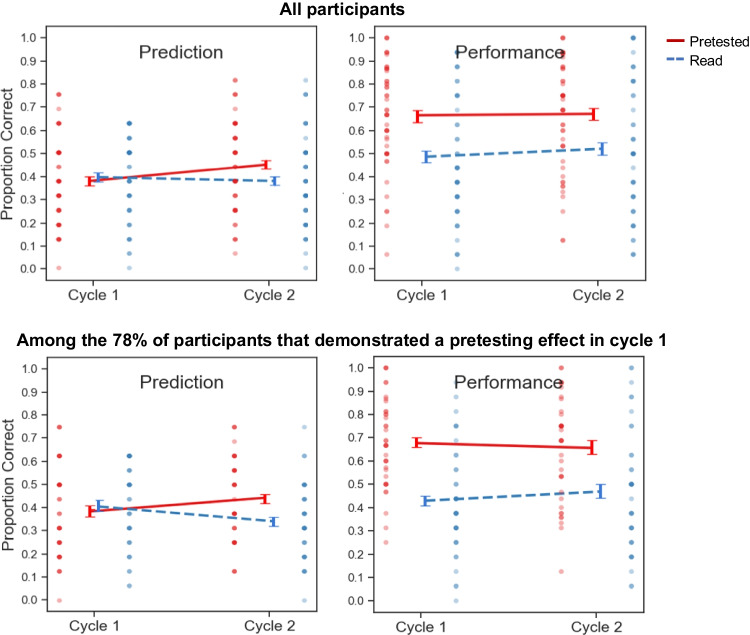
Global-differentiated predictions and criterial test performance in Experiment 4

Global-differentiated prediction and criterial test data are depicted in Fig. 6.

#### Initial test performance

As in prior experiments, participants rarely generated the correct answer to pretest trials in Cycle 1 (*M* = .040, *SE* = .0062) or in Cycle 2 (*M* = .045, *SE* = .015).

#### Criterial test performance

In Cycle 1, participants recalled a greater proportion of pretested pairs, *t*(48) = 6.87, *p* < .00001, *d* = 0.98, *BF*_*10*_ > 100. In Cycle 2, participants recalled a greater proportion of pretested pairs, *t*(48) = 5.65, *p* < .00001, *d* = 0.81, *BF*_*10*_ > 100.

#### Recall of criterial test performance

During Cycle 2, when prompted, 72% of participants were able to perfectly recall their numerical Cycle 1 criterial test performance in the pretested and read conditions. Further, 86% of participants correctly remembered the general direction of any recall difference between the pretest and read conditions correctly, irrespective of precise numerical accuracy. Thus, recall of Cycle 1 criterial test performance was relatively good (cf. Mueller et al., 2015, Experiment 4).

#### Global-differentiated predictions

A repeated-measures ANOVA on global-differentiated predictions analogous to that performed for the prior experiments revealed a significant interaction, *F*(1, 48) = 10.32, *p* = .0024, η_p_^2^ = 0.18 (see Table 1 for full results). When analysis was restricted to the 78% of participants that showed a pretesting effect on the Cycle 1 criterial test, a similar pattern held: The interaction between cycle and practice condition was significant, *F*(1, 37) = 15.43, *p* = .00036, η_p_^2^ = 0.29. These results amount to a significant replication of the critical finding of Experiment 3. Visual inspection of the results in Figure 6 confirms successful updating across cycles, although such updating still did not reflect the full magnitude of the pretesting effect.

A pair of follow-up *t* tests performed on the predictions made by participants that demonstrated a pretesting effect in Cycle 1 provided further evidence of successful updating: In Cycle 1, predictions between conditions did not significantly differ, *t*(37) = 0.89, *p =* .38, *d =* 0.14, *BF*_*01*_ = 3.95, whereas participants’ predictions for the pretested pairs were significantly higher than that for the read pairs in Cycle 2, *t*(37) = 5.23, *p <* .00001, *d =* 0.85, *BF*_*10*_ > 100. Such updating appears to have stemmed from an increase in the prediction for the pretested pairs and a corresponding decrease in the prediction for the read pairs.

When an ANOVA analogous to that described above was conducted on data restricted to the 72% of participants that were able to perfectly recall their cycle 1 criterial test performance, the interaction between cycle and practice condition was once again significant, *F*(1, 35) = 7.57, *p* = .0094, η_p_^2^ = 0.18. However, when an equivalent ANOVA was conducted on data for the 28% of participants that were unable to perfectly recall their cycle 1 criterial test performance, the interaction between cycle and practice condition was not significant, *F*(1, 12) = 2.63, *p =* .13, η_p_^2^ = .017. These results suggest that accurate memories for cycle 1 performance may be associated with successful knowledge updating (although it should also be acknowledged that these analyses were exploratory and not preregistered).

Overall, as in the prior experiment, the use of performance feedback with reminders facilitated awareness of the benefits of pretesting, and in terms of numerical magnitude, to a greater degree than in all prior experiments. The effectiveness of that feedback may have also been strengthened by participants being prompted to recall Cycle 1 performance prior to making predictions in Cycle 2.

#### Judgments of reading and pretesting

Ratings of the effectiveness of reading (*M* = 5.61, *SE* = .37) and pretesting (*M* = 6.22, *SE* = .29) did not significantly differ, *t*(48) = 1.27, *p* = .21, *d* = 0.18, *BF*_*01*_ = 3.02, which roughly matches patterns observed in the prior experiments.

## Experiment 5

The preceding experiments investigated metacognitive awareness of the pretesting effect under different forms of external support, with increasingly extensive measures intended to better fulfill the assumptions of the knowledge updating framework. Experiment 5 directly compared two of those approaches: performance feedback (as investigated in Experiment 2) and performance feedback with prediction reminders (as investigated in Experiments 3–4). In doing so, Experiment 5 addressed the replicability of patterns observed within prior experiments. To examine the stability of updating and the possibility that more experience may yield additional updating, participants also completed three learning cycles rather than two.

### Method

Experiment 5 was preregistered (https://osf.io/qad4h).

#### Participants

The target sample size of 320 participants (160 participants per group) was determined via an a priori power analysis using the *Superpower* package in R (Caldwell et al., 2020; Lakens & Caldwell, 2021), with means and standard deviations from Experiments 2 and 3 as input and assuming no correlation between measures. We sought sufficient power to detect a 2 (practice condition: read vs. pretested) × 2 (feedback group: performance feedback vs. performance feedback with reminders) interaction on global-differentiated predictions made during Cycle 2, which is where we expected to observe different judgments by practice condition. That power analysis indicated that a sample of 160 participants per group should provide 80% power to detect an interaction effect size of Cohen’s *f =* 0.16 (η_p_^2^ = 0.024) with α = 0.05.

We recruited 361 participants using Prolific Academic, with each participant randomly assigned to one of two feedback groups and awarded USD $8.75 for their participation. Data were analyzed from the 320 participants (*M*_age_ = 39.2 years, 60% female) across both groups (performance feedback only, *n* = 155; performance feedback with prediction reminders, *n =* 165) that completed the entire experiment, passed attention checks, and submitted a valid completion code. This study received ethics approval from the Psychology Department Ethics Review Committee of the National University of Singapore.

#### Design, materials, and procedure

Experiment 5 drew on the design and procedures of Experiments 2 and 3. The sole differences were that there were three training–test cycles rather than two and that each participant was randomly assigned to one of two feedback groups. The performance feedback group and the performance feedback with reminders group followed the procedures as Experiments 2 and 3, respectively. The materials were unchanged except for two additional lists (drawn from the same source; Huelser & Metcalfe, 2012) that were used in the third cycle. Finally, the same exit questions were used but posed after the third cycle.

### Results and discussion

**Fig. 7:**
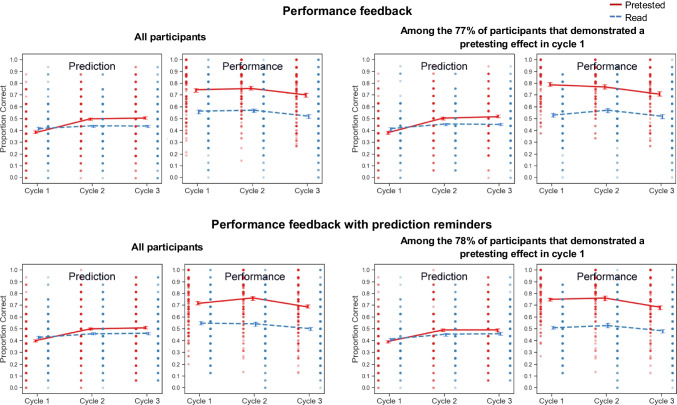
Global-differentiated predictions and criterial test performance in Experiment 5

Globally differentiated prediction and criterial test results for participants in the performance feedback and performance feedback with reminders groups are shown in the top and bottom rows of Fig. 7, respectively.

#### Initial test performance

In alignment with the patterns observed in the prior experiments, participants rarely generated the correct answer to pretest trials in both groups (across all cycles, performance feedback group: *M =* .036, *SE =* .015; performance feedback with reminders group, *M =* .040, *SE =* .0025).

#### Criterial test performance

A 3 (cycle: 1 vs. 2 vs. 3) × 2 (practice condition: read vs. pretested) × 2 (feedback group: performance feedback vs. performance feedback with reminders) mixed-factors ANOVA yielded significant main effects of cycle, *F*(2, 636) = 27.03, *p* < .0001, η_p_^2^ = 0.078, and practice condition, *F*(1, 318) = 564.15, *p* < .0001, η_p_^2^ = 0.64. The main effect of feedback group and all interactions were not significant, *F*s ≤ 2.42, *p*s *≥* .089. In both groups, participants typically recalled a greater proportion of pretested pairs in each cycle (across cycles, *t*s ≥ 11.15 *p*s < .0001, *d*s ≥ 0.87, *BF*_*10*_s > 100). Overall, there was a strong pretesting effect in both groups and throughout all cycles of the experiment.

#### Global-differentiated predictions

Per our preregistered analysis plan, we examined Cycle 2 predictions for potential differences between feedback groups. A 2 (practice condition: read vs. pretested) × 2 (feedback group: performance feedback vs. performance feedback with reminders) mixed-factors ANOVA yielded a significant main effect of practice condition, *F*(1, 318) = 51.40, *p* < .0001, η_p_^2^ = 0.14, no significant main effect of feedback group, *F*(1, 318) = 0.11, *p =* .74, η_p_^2^ = 0.00034, and no significant interaction, *F*(1, 318) = 1.52, *p =* .22, η_p_^2^ = 0.0048. When we conducted the same analysis but limited to the 248 participants that evidenced a numerical pretesting effect in Cycle 1, the same patterns were observed, with no significant main effect of feedback group or interaction (*F*s ≤ 1.87, *p*s ≤ .17). Overall, these results reflect the lack of significant differences between groups; in both groups, participants made Cycle 2 predictions that were higher for pretested than read pairs.

Thus, a pattern that might be inferred from the results of Experiments 2 versus 3—that performance feedback with prediction reminders induces more updating than performance feedback alone—was not observed in a direct comparison of the two approaches. Indeed, both approaches yielded updating in favor of pretesting in Cycle 2. Both approaches would therefore appear to be similarly capable of fostering knowledge updating (or, perhaps, adding prediction reminders to performance feedback may not necessarily yield substantially greater updating).

To determine whether the patterns observed in Experiments 2 and 3 replicated, we also conducted repeated-measures ANOVAs limited to data from each feedback group (see Table 5 for full results), followed by *t* tests for data from each cycle. In summary, the overall patterns observed in both experiments replicated: In both groups, predictions did not favor either condition in Cycle 1 (*t*s ≤ 1.92, *p*s ≥ .054, *d*s ≤ 0.17, *BF*_*10*_s ≤ 0.60), then switched to favoring pretesting in Cycles 2 and 3 (*t*s ≥ 4.15, *p*s < .0001, *d*s ≥ 0.36, *BF*_*10*_s > 100). In a supplementary analysis (see Appendix), we also examined whether the amount of updating increased from Cycle 2 to 3 and found that it only did so for the case of participants that exhibited a numerical pretesting effect in the performance feedback group.

**Table 5:** Outcomes of group-level analyses of variance for globally-differentiated predictions in Experiment 5

	*df*	*F*	*p*	η_p_^2^
Performance feedback group: 3 (cycle: 1 vs. 2 vs. 3) × 2 (practice condition: read vs. pretested)				
Main effect of cycle	2, 230	12.72	<.0001***	0.010
Main effect of practice condition	1, 115	28.03	<.0001***	0.20
Interaction	2, 230	21.33	<.0001***	0.16
Performance feedback with reminders group: 3 (cycle: 1 vs. 2 vs. 3) × 2 (practice condition: read vs. pretested)				
Main effect of cycle	2, 264	15.93	<.0001***	0.11
Main effect of practice condition	1, 132	14.54	.00021***	0.099
Interaction	2, 264	14.48	<.0001***	0.099

*** indicate *p* values < .001

#### Judgments of reading and pretesting

In the performance feedback group, ratings of the effectiveness of pretesting (*M =* 7.16, *SE =* .13) were significantly higher than reading (*M =* 5.60, *SE =* .16), *t*(164) = 6.00, *p < .*0001 , *d =* .47, *BF*_*10*_ > 100. In the performance feedback with reminders group, ratings of the effectiveness of pretesting (*M =* 7.11, *SE =* .15) were also significantly higher than reading (*M =* 5.93, *SE =* .16), *t*(154) = 8.29, *p < .*0001 , *d =* .67, *BF*_*10*_ > 100. These results represent the first case wherein pretesting received significantly higher ratings than reading and suggest that preference for pretesting and awareness of its greater effectiveness can manifest after more extensive experience.

## General discussion

The current study reveals insights into the persistence of inaccurate metacognitive beliefs about pretesting, the need for support to overcome those inaccurate beliefs, and conditions under which awareness of the pretesting effect can occur. Repeated experience with pretesting did not cause learners to spontaneously update their beliefs (Experiment 1). That result led us to implement several forms of external support that were designed to fulfill key assumptions of the knowledge updating framework (Dunlosky & Hertzog, 2000) and foster successful updating. Providing learners with feedback on their criterial test performance was at least partially effective (Experiments 2 and 5). When that feedback included reminders of learners’ original predictions (Experiments 3–5), knowledge updating was also observed. Moreover, accurate knowledge of criterial test performance was associated with successful updating (Experiment 4). The addition of a third cycle in Experiment 5—which was, to our knowledge, the first investigation of knowledge updating across more than two training–test cycles— yielded preference and effectiveness ratings that favored pretesting and mixed results for further updating of global-differentiated predictions in Cycles 2 versus 3 (see Appendix).

Overall, the present findings reveal that fostering metacognitive awareness of the pretesting effect is possible. Doing so, however, requires support in the form of performance feedback, feedback with prediction reminders, and/or recalling criterial test performance (cf. Pressley et al., 1984; Ringel & Springer, 1980; Tullis et al., 2013). We next interpret our results in the context of the knowledge updating framework.

### Conditions for knowledge updating of the pretesting effect

Learners do not automatically develop awareness of the pretesting effect through experience. In all experiments, most participants exhibited a pretesting effect on both criterial tests (first cycle, 70%–82% of participants; second cycle, 60%–86%), thus meeting the effectiveness assumption of Dunlosky and Hertzog (2000), but there was no corresponding knowledge updating in the absence of external support (Experiment 1). Why did that lack of awareness, which extends the results of prior research, persist? One possibility is that remembering the technique that was used to learn each item and then generalizing across items is too burdensome (thus failing to meet the monitoring assumption). This burden could have resulted from the difficulty of keeping track of when pretested and read items were presented in a randomized order during study and/or test (e.g., Hertzog et al., 2009; Price et al., 2008) or discriminating between small differences in recall between items learned with each strategy (Rivers et al., 2022). Consequently, learners were unaware of the differential effectiveness of the two techniques. Another nonexclusive possibility is that preexisting biases against pretesting (Pan, Sana, Samani, et al., 2020) could influence multiple aspects of the knowledge updating process (i.e., relating to the monitoring, updating, and/or utilization assumptions).

To increase participants’ awareness of the pretesting effect, from Experiment 2 onward we provided performance feedback after each criterial test. In the case of retrieval practice versus restudy, Tullis et al. (2013) used performance feedback to alleviate the metacognitive burden imposed by trial-level randomization of items learned with each strategy. In Experiment 2 and in the corresponding group in Experiment 5, performance feedback was partially effective and fully effective at eliciting updating, respectively. Informing learners about their performance on pretested versus read items evidently helped fulfill the monitoring assumption, which in turn helped fulfill the updating and utilization assumptions, leading to updating. The amount of updating in Experiment 2, however, was relatively limited. An explanation for that result is that learners did not apply knowledge of performance differences when making Cycle 2 predictions (a so-called utilization deficit in judgments; Mueller et al., 2015); alternatively, those results may have reflected random chance. Ultimately, given that successful updating was clearly observed when the same procedures were applied with a much larger sample in Experiment 5, we suspect that performance feedback should be sufficient, in most circumstances, to promote metacognitive awareness of the pretesting effect.

In Experiments 3 and 4 (and in the corresponding group in Experiment 5), the use of prediction reminders to draw learners’ attention to their mistaken metacognitive judgments—and in turn, possibly flawed underlying beliefs—also helped foster awareness of the pretesting effect. In those experiments, learners were not just made aware that pretesting is more effective than reading (as was the case with performance feedback alone); they also saw that they did not anticipate the mnemonic benefit of pretesting. With performance-prediction discrepancies in mind, learners adjusted their metacognitive judgments accordingly, at least with respect to global-differentiated predictions.

It should be noted, however, that the results of Experiment 5 reveal that prediction reminders do not yield greater updating than performance feedback alone. One important distinction to consider here is that in the case of performance feedback, learners contrasted their performance in the pretested and read conditions (in the attention check question posed immediately afterwards), whereas for performance feedback with reminders, they did not—instead, they contrasted their predictions with performance separately for the pretested and read pairs. We suspect that the reminders may have been more effective if learners were asked to directly contrast pretesting and reading. As a broader point, the potential role of these questions in fostering knowledge updating, which reinforced the feedback provided in Experiments 2–5, should not be discounted.

In Experiment 4, the addition of recall prompts, which required learners to remember their performance on the first criterial test for pretested versus read items, also appeared to facilitate knowledge updating. Having learners recall their performance may remind them about the differential effectiveness of strategies (which relieves the burden of having to monitor strategy effectiveness during the criterial test), plus make knowledge about the differential effectiveness of strategies salient at the time of predictions (helping fulfill the utilization assumption). Most participants were able to recall their performance accurately, and among those that did, successful updating occurred. Note, however, that the benefits of recall prompts were not observed in isolation. Indeed, the knowledge updating in Experiment 4 may have stemmed from the combined impact of all the methods of external support that were used, namely performance feedback, prediction reminders, and recall prompts.

In Experiments 1–4, learners did not express a strong preference for pretesting over reading in a hypothetical scenario. Their effectiveness ratings for both techniques also did not significantly differ (although numerically they increasingly favored pretesting across experiments). In Experiment 5, however, learners expressed a strong preference for pretesting and gave it higher effectiveness ratings than for reading. These results suggest that more extensive experience—that is, three cycles rather than two—can indeed yield greater metacognitive awareness of the pedagogical utility of pretesting. Two cycles of experience may be insufficient to achieve that level of awareness (although any comparisons across experiments are tentative); under those circumstances, learners may still rely on declarative knowledge about specific strategies (cf. Price et al., 2008), greater comfort or prior experience with reading (Bjork et al., 2013), or the belief that reading is more effective outside of the current experimental context. Selective updating for some types of judgments, but not others, has also been observed in prior knowledge updating research (e.g., Mueller et al., 2015).

### Implications for knowledge updating and applications of pretesting

The current results reveal limitations of two approaches that have facilitated knowledge updating for other strategies (e.g., keyword method versus rote repetition). These approaches include delaying metacognitive judgments after strategies have been used (e.g., Pressley et al., 1984; cf. Shaughnessy, 1981) and having learners take a criterial test (e.g., Pressley et al., 1984). Although both approaches were included in this study, they were inadequate to facilitate knowledge updating. The threshold for metacognitive awareness of the pretesting effect is evidently higher than that for some other learning techniques.

Practically speaking, the current study informs several tentative recommendations for enhancing awareness of the pretesting effect (although it should be acknowledged that learners may be learning different kinds of materials than paired associates). First, learners should not be expected to spontaneously develop awareness through repeated experience alone; unlike some other learning techniques, the benefits of pretesting are not typically self-evident. Second, our data suggest that effective measures for promoting such awareness include providing performance feedback showing the effectiveness of pretesting, highlighting discrepancies between learners’ expectations (or beliefs) and the actual effectiveness of pretesting (for related discussion, see McDaniel & Einstein, 2020), and doing so over multiple rounds of training and testing. One component of an intervention aimed at increasing learners’ use of pretesting during learning might involve a classroom demonstration in which learners repeatedly use pretesting and reading to learn word pairs, make predictions, take a memory test, and then compare their predictions to their performance (cf. Einstein et al., 2012). An important aspect of such a demonstration is that the instructor can control factors such as study time and test difficulty, thereby isolating the learning strategy as the cause of performance differences and aiding in accurate self-reflection. Our data suggests that such a demonstration is likely to foster appreciation for the benefits of pretesting, and prompting learners to reflect on their performance can promote the use of effective learning strategies (e.g., Berthold et al., 2007).

### Future research directions

Follow-up research might investigate circumstances that were not fully addressed in the foregoing experiments. Given that knowledge updating remained incomplete relative to actual performance, even in the final experiment, yet other approaches to promote metacognitive awareness (such as segregating read versus pretested items into separate activity periods as in Price et al., 2008; see also Yan et al., 2016) could be investigated. Additionally, more educationally relevant materials (e.g., expository texts, lecture videos) could be explored across longer retention intervals to determine generalizability (especially given that materials that are more commonly used in educational contexts, and for which pretesting has been demonstrated to enhance learning, were not used in the present experiments), and methodological variations on the pretesting paradigm could be considered. Finally, the potential role of individual differences in the effects of pretesting on learning (e.g., across experiments, 53%–74% of participants that exhibited a pretesting effect in Cycle 1 also did so in Cycle 2), and how those differences might impact metacognition, could be investigated. Ultimately, further work on this topic stands to reveal additional insights into learners’ metacognitive beliefs about pretesting and inform the development of interventions aimed at facilitating knowledge updating of effective learning techniques. These interventions, in turn, will foster improvements in self-regulated learning and academic achievement.

## Appendix

### Supplementary analysis of knowledge updating in Cycles 2 and 3 of Experiment 5

To examine possible changes in the amount of updating from Cycles 2 to 3 in Experiment 5, we conducted an exploratory 2 (cycle: 2 vs. 3) × 2 (practice condition: read vs. pretested) repeated-measures ANOVA separately on global-differentiated predictions for each feedback group. In the analysis for the performance feedback group, only the main effect of practice condition was significant, *F*(1, 154) = 48.60, *p* < .0001, η_p_^2^ = 0.24. The main effect of cycle and the interaction between cycle and practice condition were not significant (*p ≥* .43). When the analysis was restricted to participants that demonstrated a numerical pretesting effect in Cycle 1, however, there was a significant interaction between cycle and practice condition, *F*(1, 115) = 5.08, *p* = .026, η_p_^2^ = 0.42. That result suggests that for those participants, the amount of updating improved from Cycle 2 to Cycle 3. That pattern, however, was not apparent in the aforementioned analysis involving the entire group (see Fig. 7).

In the analysis for the performance feedback with reminders group, only the main effect of practice condition was significant, *F*(1, 164) = 31.04, *p* < .0001, η_p_^2^ = 0.16. The main effect of cycle and the interaction between cycle and practice condition were not significant (*p ≥* .50). When the analysis was restricted to participants that demonstrated a numerical pretesting effect in Cycle 1, the same pattern was observed, with only a significant main effect of practice condition, *F*(1, 132) = 30.57, *p* < .0001, η_p_^2^ = 0.19, and no significant main effect of cycle or cycle by practice condition interaction (*p ≥* .39). Thus, no evidence was found for improved updating from Cycle 2 to Cycle 3 in that group.

## Footnotes

1 Although the pretested condition had longer total trial times, equivalent time was spent viewing the correct answer in both the read and pretested conditions; see Kornell et al. (2009) for evidence that the pretesting effect survives under equivalent total trial times, and Hays et al. (2013) for discussion of feedback timing.
